# Cystic Artery Pseudoaneurysm: Current Review of Aetiology, Presentation, and Management

**DOI:** 10.1155/2021/4492206

**Published:** 2021-11-24

**Authors:** Seyed Mohammad Javad Taghavi, Mahendra Jaya kumar, Ramesh Damodaran Prabha, Harald Puhalla, Craig Sommerville

**Affiliations:** ^1^Gold Coast University Hospital, General Surgery, Gold Coast 4215, QLD, Australia; ^2^University of New South Wales, Liverpool Hospital, Liverpool 2170, NSW, Australia

## Abstract

**Background:**

Cystic artery pseudoaneurysms are rare. Most commonly, they occur secondary to acute cholecystitis or after a cholecystectomy. Complications include haemobilia, biliary obstruction, and haemorrhage. Given the rarity and associated morbidity, a high index of suspicion is required. This article reviews the current literature on cystic artery pseudoaneurysms to investigate its aetiology, clinical presentation, and management options.

**Methods:**

A broad search of the Medline and PubMed databases was carried through. All peer reviewed literatures published in the English language between 1991 and 2020 with keywords “cystic” and “artery” and “pseudoaneurysm” in the title were selected for review. No further exclusion criteria; all studies yielded from the search were included in the results of this review. Additionally, we present a case of cystic artery pseudoaneurysm treated at our centre and included this in our analysis.

**Results:**

Sixty-seven case reports were found between 1991 and 2020. Aetiologies: Aetiology of cystic artery pseudoaneurysm was found to be cholecystitis in 41 instances (61.2%), cholecystectomy in 18 instances (26.8%), idiopathic in 6 instances (8.9%) cholelithiasis in 1 instance (1.5%), and pancreatitis in 1 instance (1.5%). Complications: Fifty-two cases were complicated by haemobilia (77.6%), 36 by anaemia (53.7%), 25 by biliary obstruction (37.3%), 13 by haemodynamic shock (19.4%), 9 by haemoperitoneum (13.4%), and 6 by contained rupture (8.9%). Most commonly, patients had two or more of these complications. Management: Forty-four patients were managed with endovascular embolisation (65.7%), 21 with endoscopic intervention (31.3%), 18 with open cholecystectomy (26.9%), 13 with laparoscopic cholecystectomy (19.4%), and 6 with pseudoaneurysm ligation (9%). Delayed presentation postcholecystectomy ranged from 8 days to 3 years.

**Conclusions:**

Cystic artery pseudoaneurysms are rare complications of a common operation. The most common clinical presentation is haemobilia, which can be difficult to diagnose clinically. A high index of suspicion and prompt investigation with targeted imaging and intervention is required. This is especially pertinent in gastrointestinal bleeding postlaparoscopic cholecystectomy as a missed diagnosis could cause significant morbidity.

## 1. Background

Laparoscopic cholecystectomy (LC) is a routine surgical procedure for the treatment of gallbladder pathology. In Australia, there are an approximate 50,000 annual hospitalisations for LC [1]. The incidence of intraabdominal vascular complications from LC is uncertain. Published data report incidences as low as 0.25% and as high as 7%, although there is variation in the patient population and investigation methods between studies [2, 3]. Cystic artery pseudoaneurysm (CAP) is a rare vascular complication of LC. Other aetiologies for CAP include cholecystitis, cholelithiasis, and idiopathic CAP [4–6]. The complications of CAP include haemobilia, anaemia, biliary obstruction, and haemoperitoneum and haemodynamic shock [3]. The classical clinical syndrome of haemobilia is described by Quinke's triad: jaundice, right upper quadrant abdominal pain, and upper gastrointestinal bleeding, present in approximately 22–35% of cases [6]. In this article, we present a thorough review of the current published literature to identify the aetiology, clinical presentation, and management of CAP. We also present a case of CAP rupture postcholecystectomy managed at our centre and included this in our data analysis.

## 2. Methods

A broad search of the Medline and PubMed databases was carried out. All peer reviewed literatures published in the English language between 1991 and 2020 with the keywords “cystic” and “artery” and “pseudoaneurysm” in title were selected for review. No further exclusion criteria; all studies yielded from our search were ultimately included in the results of this review. Additionally, we present, with written consent from the patient, a case of cystic artery pseudoaneurysm treated at our centre and include this in our analysis.

## 3. Results

### 3.1. Case Report

**Figure 1:**
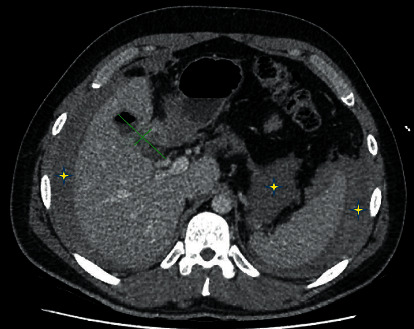
Computed tomography scan of the abdomen in the axial plane showing a gallbladder fossa collection (green marks), retroperitoneal, perihepatic, and perisplenic haematomas (yellow stars).

**Figure 2:**
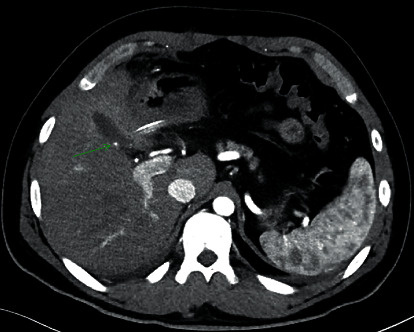
Contrast-enhanced computed tomography scan of the abdomen in the axial plane showing a cystic artery pseudoaneurysm (green arrow).

A 29-year-old male underwent an uneventful laparoscopic cholecystectomy for recurrent biliary colic. There was no aberrant anatomy noted, and intraoperative cholangiography was normal. On postoperative day 8, he represented to the emergency department with increasing abdominal pain. On examination, he was tachycardic with a distended tender abdomen, and a periumbilical haematoma was noted. Initial investigation showed a haemoglobin of 136 g/L, a white cell count of 16.5 × 10^9^/L, and normal liver function and coagulation studies. Initial contrast CT of the abdomen (Figure 1) showed a gas and fluid collection at the gallbladder fossa and large volume haemoperitoneum. No active contrast extravasation was detected. The patient proceeded to laparoscopic washout, and 1.5 L of blood and clot was evacuated from all quadrants. It was noted that the cystic artery and duct clips were intact. No obvious bleeding point was identified. Two 19F Blake drains were placed in the gallbladder fossa and pelvis. The patient recovered uneventfully postoperatively, and the drains were removed on postoperative day 4. Interval CT angiography was performed on postoperative day 5 to further investigate the cause of delayed bleeding. This showed resolution of haemoperitoneum but revealed a 3 mm arterial hyperenhancement in the gallbladder fossa consistent with a pseudoaneurysm of the cystic artery (Figure 2). Follow-up digital subtraction angiography showed a cystic artery pseudoaneurysm and another pseudoaneurysm in the right hepatic artery branch to segment 4a. Both pseudoaneurysms were successfully embolised with a combination of Tornado® Coils (Cook Medical, Ireland) and Gelfoam® (Pfizer, USA). The patient was discharged on postoperative day 9 and was well at two-month follow-up. No further surveillance was required.

### 3.2. Literature Review

Sixty-seven case reports of CAP were identified between 1991 and 2020, including the patient treated at our centre. The mean age at diagnosis was 60 years, with a range of 23–91 years. Forty-five of 67 patients (67%) were male.

#### 3.2.1. Aetiologies

Of the 67 cases of CAP reported, 4 cases were secondary to cholecystitis (61.2%) [7–44], 18 followed cholecystectomy (26.8%) [45–61], 6 were idiopathic (8.9%) [5, 62–65], 1 correlated with cholelithiasis (1.5%) [4], and 1 with pancreatitis (1.5%) [66]. Of the patients with CAP postcholecystectomy, the median postoperative time to clinical presentation was 50 days, with a range of eight days (in our patient) to three years [52].

#### 3.2.2. Clinical Presentations

Fifty-eight of 67 patients (86.5%) presented with right upper quadrant pain. Fifty-two presented with haemobilia (77.6%). Seventy-seven percent of the patients who presented with haemobilia (*n* = 40/52) had clinical upper gastrointestinal bleeding (haematemesis or melena) and subsequent endoscopy confirming bleeding from the ampulla of Vater. The remaining 23% of patients with haemobilia (*n* = 12/52) were diagnosed on endoscopic retrograde cholangiopancreatography (ERCP), intraoperatively during laparoscopy or laparotomy, or on insertion of a percutaneous cholecystostomy tube as part of management for cholecystitis.

Twenty-five of 67 patients (32.8%) presented with obstructive derangement of liver function tests. Of these, 22 were secondary to haemobilia and 3 had biliary obstruction due to direct compression of the bile duct by CAP. Nineteen of 22 patients with biliary obstruction due to haemobilia had clinical jaundice. All 3 of the patients with biliary obstruction due to direct compression of the bile duct were jaundiced. Overall, Quinke's triad was present in 11 of 52 (21.2%) patients with haemobilia.

Fifteen of the 67 (22.4%) patients had a ruptured CAP at clinical presentation, 6 of whom had a contained rupture and 9 who had haemoperitoneum. Overall, 36 of 67 patients (53.7%) were anaemic and 13 of 67 patients (19.4%) had haemodynamic shock at clinical presentation. The median and mode number of the aforementioned presenting problems in each of the 67 patients was 3, with a range of 0–6.

#### 3.2.3. Diagnostic Modalities

Forty-six of 67 patients (68.6%) were diagnosed with CAP based on the results of arterial phase contrast-enhanced computed tomography (CT) scans. Thirteen patients (19.4%) were diagnosed with digital subtraction angiography. Colour Doppler ultrasound and magnetic resonance imaging were each used to reach diagnosis in three patients (4.4%). Two patients (3%) had an intraoperative finding of CAP at cholecystectomy for cholecystitis.

#### 3.2.4. Management Options

Forty-four patients were treated with endovascular embolisation of CAP (65.7%). Of these 44 procedures, 4 were unsuccessful, with 3 of these proceeding to surgery and one being managed successfully with a reattempted endovascular approach. Including the 3 patients for whom endovascular embolisation was unsuccessful, a total of 6 patients (9%) were managed with laparotomy and ligation of the cystic artery proximal to the aneurysm. Two patients were treated with percutaneous thrombin injection (3%). Of these, one procedure was unsuccessful and was subsequently successfully managed with endovascular embolisation. 21 patients (31.3%) required ERCP. 31 patients underwent cholecystectomy as part of their treatment (46.3%). Of the patients who had a cholecystectomy, 12 were following successful endovascular embolisation of the pseudoaneurysms and 19 had cholecystectomy with proximal ligation or clipping of the cystic artery as the only management modality. No mortalities were reported.

Table 1 provides the aetiologies, diagnostic imaging modalities, clinical presentations, and management of CAP in the cases reported.

## 4. Discussion

CAP is a rare phenomenon with significant risk of morbidity in the form of biliary obstruction and bleeding. Most commonly, it occurs secondary to acute cholecystitis or after cholecystectomy. The pathophysiology of CAP in cholecystitis is not clear, though it is likely related to inflammatory damage and weakening of the adventitia with subsequent pseudoaneurysm formation [3, 9]. In addition to this, surgery may contribute further to the development of CAP due to vascular erosion from manipulation, clip application, or thermal injury, although the incidence of vascular injury in cholecystectomy is low (0.2–0.5%) [52, 56]. In our reported case, we suspect that the right hepatic artery pseudoaneurysm is explained by some degree of regional inflammation secondary to cholelithiasis, and the CAP is explained by the same in addition to the effect of surgery.

Haemobilia is by far the most common clinical presentation of CAP, with the most common symptom of this being clinical upper gastrointestinal bleeding in the form of haematemesis or melena. Haemobilia can be difficult to diagnose clinically without a high index of suspicion and prompt investigation with targeted imaging. The reported incidence of Quinke's triad in patients with haemobilia is 22–35% [6]. Our review supports this statistic as the triad was present in 21.2% of the cases of CAP with haemobilia. Furthermore, there is a significant variance in the time from cholecystectomy to clinical presentation with a symptomatic CAP in patients where cholecystectomy is the aetiology. This makes a high index of suspicion particularly pertinent in the postcholecystectomy cohort. It is also noteworthy that a symptomatic CAP has been reported up to three years postcholecystectomy [52].

Overall, the morbid load of CAP when symptomatic is high, with most patients suffering at least three of the following problems at clinical presentation: haemobilia, biliary obstruction, anaemia, and haemoperitoneum or haemodynamic shock. It is important to note, despite its morbid load, that CAP is uncommon, and therefore, the astute clinician must pay mind to assessing the patient with due attention to differential causes of these findings.

The most common diagnostic modality for CAP is an arterial phase contrast-enhanced CT scan, which is a readily available imaging modality in most treatment centres. Colour Doppler ultrasound and MRI imaging are options for patients with contraindications to the use of intravenous contrast agents.

Endovascular embolisation was the most common choice of controlling CAP in our review, with an adequate rate of success on the first attempt in the majority of patients. Surgery in the form of cholecystectomy or vessel ligation would be most likely indicated in cases where the endovascular approach is unsuccessful.

Though the findings are of interest in light of the rarity of this pathology, our review is limited by its retrospective nature and broad inclusion criteria. There is no clear cause and effect relationship demonstrated by the aetiologies listed, and the therapeutic benefit of the management modalities compared to one another cannot be assessed. Perhaps, the strongest correlation that can be made is in regard to the way CAP clinically presents, as the clinical presentations can be directly corroborated with gold-standard confirmatory diagnostic imaging.

## 5. Conclusion

Although CAP is a rare phenomenon, it occurs as a complication of one of the most common surgical procedures performed worldwide, and it carries with it significant associated morbidity. It is therefore pertinent to the surgeon to be aware of its clinical presentation, diagnosis, and management options. A high index of suspicion and prompt investigation is suggested in patients with cholecystitis or previous cholecystectomy who present with upper gastrointestinal bleeding, anaemia, and biliary obstruction.

## Figures and Tables

**Table 1:** A summary of the aetiologies, diagnostic imaging modalities, presentations, and management of CAP.

Aetiology		Diagnostic imaging modality		Complications		Management	
Cholecystitis	41 (61.2%)	CT^†^	46 (68.6%)	Haemobilia	52 (77.6%)	Endovascular embolisation	44 (65.7%)
Cholecystectomy	18 (26.8%)	DSA^‡^	13 (19.4%)	Haemobilia + Quinke's triad^*∗*^	11 (16.5%)	Percutaneous thrombin injection	2 (3%)
Idiopathic	6 (8.9%)	US^§^	3 (4.4%)	Haemobilia with haematemesis or melena	39 (58.3%)	ERCP^¶^	21 (31.3%)
Cholelithiasis	1 (1.5%)	MRI^||^	3 (4.4%)	Haemobilia with biliary obstruction	22 (32.8%)	Cholecystectomy	31 (46.3%)
Pancreatitis	1 (1.5%)			Direct biliary obstruction	3 (4.5%)	Laparotomy, vessel ligation	6 (9%)
				Contained rupture	6 (8.9%)		
				Rupture with haemoperitoneum	9 (13.4%)		
				Haemodynamic shock	13 (19.4%)		

The number of patients in each category is presented, with a percentage in relation to the total of 67 reported cases of CAP in brackets. ^†^Contrast-enhanced computed tomography. ^‡^Digital subtraction angiography. ^§^Colour Doppler ultrasound. ^||^Magnetic resonance imaging. ^¶^Endoscopic retrograde cholangiopancreatography. ^*∗*^Haemobilia + haematemesis or melena + biliary obstruction.

## Data Availability

The data used to support the findings of this study are included within the article and the references provided.

## References

[1] (2017). https://www.safetyandquality.gov.au/sites/default/files/migrated/4.4-Laparoscopic-cholecystectomy.pdf.

[2] Tzovaras G., Dervenis C. (2006). Vascular injuries in laparoscopic cholecystectomy: an underestimated problem. *Digestive Surgery*.

[3] Machado N., Al-Zadjali A., Kakaria A., Younus S., Rahim M., Al-Sukaiti R. (2017). Hepatic or cystic artery pseudoaneurysms following a laparoscopic cholecystectomy: literature review of aetiopathogenesis, presentation, diagnosis and management. *Sultan Qaboos University medical journal*.

[4] Trombatore C., Scilletta R., Bellavia N. (2017). Acute hemobilia from a pseudoaneurysm of the cystic artery arising from the left hepatic artery: case report and literature review. *International Journal of Surgery Case Reports*.

[5] Totolici B. D., NeamŢu C., Bodog F. D. (2017). Hemobilia by idiopathic aneurysm of cystic artery, fistulized in the biliary ways - clinical case. *Romanian journal of morphology and embryology = Revue roumaine de morphologie et embryologie*.

[6] Berry R., Han J., Kardashian A. A., LaRusso N. F., Tabibian J. H. (2018). Hemobilia: etiology, diagnosis, and treatment. *Liver Research*.

[7] Hall T. C., Sprenger De Rover W., Habib S., Kumaran M. (2016). Cystic artery pseudoaneurysm secondary to acute cholecystitis: an unusual cause for haemobilia. *BJR Case Reports*.

[8] Gapp J., Mukherjee T., Gross J., Sirineni G., Abdalla A. (2018). Ruptured cystic artery pseudoaneurysm as a cause of cholestasis. *American Journal of Gastroenterology*.

[9] Loizides S., Ali A., Newton R., Singh K. K. (2015). Laparoscopic management of a cystic artery pseudoaneurysm in a patient with calculus cholecystitis. *International Journal of Surgery Case Reports*.

[10] Praveen Kumar Sunkara P. R. V., Shah P. K., Rakshit K., Choudhary S. R., Bohidar N. P., Dubey S. K. (2017). Rupture of cystic artery pseudoaneurysm: a rare complication of acute cholecystitis. *Indian Journal of Surgery*.

[11] Proença A. L., Veloso Gomes F., Costa N., Bilhim T., Luz J. H., Coimbra E. (2019). Transarterial embolization of iatrogenic cystic artery pseudoaneurysm. *GE—Portuguese Journal of Gastroenterology*.

[12] Fujimoto Y., Tomimaru Y., Hatano H. (2018). Ruptured cystic artery pseudoaneurysm successfully treated with urgent cholecystectomy: a case report and literature review. *American Journal of Case Reports*.

[13] Komatsu (2011). Report of a case: pseudoaneurysm of the cystic artery with hemobilia treated by arterial embolization. *Journal of Medical Cases*.

[14] Kuzman M., Adiamah A., Higashi Y., Gomez D. (2018). Rare Case of Cystic Artery Pseudoaneurysm. *BMJ Case Reports*.

[15] Zucker B., Walsh U., Nott D. (2017). Laparoscopic treatment of cystic artery pseudoaneurysm in the presence of calculous cholecystitis. *Annals of the Royal College of Surgeons of England*.

[16] Kulkarni V., Deshmukh H., Gupta R. (2014). Pseudoaneurysm of anomalous cystic artery due to calculous cholecystitis. *Case Reports*.

[17] Tanaka T., Takakura K., Maruyama Y. (2019). Hemobilia derived from cystic artery pseudoaneurysm. *Case Reports In Gastroenterology*.

[18] Akatsu T., Tanabe M., Shimizu T. (2007). Pseudoaneurysm of the cystic artery secondary to cholecystitis as a cause of hemobilia: report of a case. *Surgery Today*.

[19] Maeda A., Kunou T., Saeki S. (2002). Pseudoaneurysm of the cystic artery with hemobilia treated by arterial embolization and elective cholecystectomy. *Journal of Hepato-Biliary-Pancreatic Surgery*.

[20] Nakajima M., Hoshino H., Hayashi E. (1996). Pseudoaneurysm of the cystic artery associated with upper gastrointestinal bleeding. *Journal of Gastroenterology*.

[21] Shimada K., Sakamoto Y., Esaki M., Kosuge T. (2008). Pseudoaneurysm of the cystic artery associated with xanthogranulomatous cholecystitis. *Digestive Surgery*.

[22] Siddiqui N. A., Chawla T., Nadeem M. (2011). Cystic artery pseudoaneurysm secondary to acute cholecystitis as cause of haemobilia. *Case Reports*.

[23] Sousa H. T., Amaro P., Brito J. (2009). Hemobilia due to pseudoaneurysm of the cystic artery. *Gastroenterologie Clinique et Biologique*.

[24] Liang X., Lü J. M., Meng N., Jin R. A., Cai X. J. (2013). Hemorrhagic shock caused by rupture of cystic artery pseudoaneurysm secondary to calculous cholecystitis. *Chinese Medical Journal*.

[25] Ghoz A., Kheir E., Kotru A. (2007). Hemoperitoneum secondary to rupture of cystic artery pseudoaneurysm. *Hepatobiliary and Pancreatic Diseases International: HBPD INT*.

[26] Pérez-Castrillón J. L., Mendo M., Calero H. (2006). Hemorrhage into the gallbladder caused by pseudoaneurysm of the cystic artery. *Endoscopy*.

[27] Fung A., Vosough A., Olson S., Aly E., Binnie N. (2013). An unusual cause of acute internal haemorrhage: cystic artery pseudoaneurysm secondary to acute cholecystitis. *Scottish Medical Journal*.

[28] Mokrane F.-Z., Alba C. G., Lebbadi M. (2013). Pseudoaneurism of the cystic artery treated with hyperselective embolisation alone. *Diagnostic and interventional imaging*.

[29] Lee J. W., Kim M. Y., Kim Y. J., Suh C. H. (2006). CT of acute lower GI bleeding in chronic cholecystitis: concomitant pseudoaneurysm of cystic artery and cholecystocolonic fistula. *Clinical Radiology*.

[30] Desai A. U., Saunders M. P., Anderson H. J., Howlett D. C. (2010). Successful transcatheter arterial embolisation of a cystic artery pseudoaneurysm secondary to calculus cholecystitis: a case report. *Journal of Radiology Case Reports*.

[31] Alis D., Ferahman S., Demiryas S., Samanci C., Ustabasioglu F. E. (2016). Laparoscopic management of a very rare case: cystic artery pseudoaneurysm secondary to acute cholecystitis. *Case Reports In Surgery*.

[32] Joyce M. R., Donnolly M., O’Shea L., Jeffers M., O’Riordain D. (2006). Pseudoaneurysm of the cystic artery: a diagnostic dilemma and rare cause of haemobilia. *Irish Journal of Medical Science*.

[33] Gutiérrez G., Ramia J. M., Villar J., Garrote D., Ferron A., Ruiz E. (2004). Cystic artery pseudoaneurism from an evolved acute calculous cholecystitis. *The American Journal of Surgery*.

[34] Strickland S. K., Khoury M. B., Kiproff P. M., Raves J. J. (1991). Cystic artery pseudoaneurysm: a rare cause of hemobilia. *CardioVascular and Interventional Radiology*.

[35] Glaysher M. A., Cruttenden-Wood D., Szentpali K. (2014). A rare cause of upper gastrointestinal haemorrhage: ruptured cystic artery pseudoaneurysm with concurrent cholecystojejunal fistula. *International Journal Of Surgery Case Reports*.

[36] She W., Tsang S., Poon R., Cheung T. (2017). Gastrointestinal bleeding of obscured origin due to cystic artery pseudoaneurysm. *Asian Journal of Surgery*.

[37] Ahmed I., Tanveer U. H., Sajjad Z., Munazza B., Azeem U. D., Basit S. (2010). Cystic artery pseudo-aneurysm: a complication of xanthogranulomatous cholecystitis. *British Journal of Radiology*.

[38] Maddineni S., D Lim M. M., McCabe S., Rozenblit G. (2017). Transcatheter embolization of a cystic artery pseudoaneurysm in a cirrhotic patient with perforated acute cholecystitis. *Indian Journal of Radiology and Imaging*.

[39] Tapnio R. H., Kolber M. K., Shukla P. A., Berkowitz E. (2017). Transcatheter embolization of cystic artery pseudoaneurysms secondary to acute cholecystitis. *Vascular and Endovascular Surgery*.

[40] Suzuki S., Saito Y., Nakamura K. (2013). Unruptured cystic artery pseudoaneurysm accompanied by Mirizzi syndrome: a report of a case. *Clinical journal of gastroenterology*.

[41] Anand U., Thakur S. K., Kumar S., Jha A., Prakash V. (2011). Idiopathic cystic artery aneurysm complicated with hemobilia. *Annals of Gastroenterology*.

[42] Barba C. A., Bret P. M., Hinchey E. J. (1994). Pseudoaneurysm of the cystic artery: a rare cause of hemobilia. *Canadian journal of surgery. Journal canadien Carey*.

[43] Ghosh Chatterjee J., Das S., Konar A. (2019). Cystic artery pseudoaneurysm and severe cholangitis - a rare association - a case report. *Acta Scientific Gastrointestinal Disorders*.

[44] Hague J., Brennand D., Raja J., Amin Z. (2010). Cystic artery pseudoaneurysms in hemorrhagic acute cholecystitis. *CardioVascular and Interventional Radiology*.

[45] Liu B., Lewis A. R., Ward T. J. (2016). Cystic artery pseudoaneurysm. *Journal of Vascular and Interventional Radiology*.

[46] Rege S. A., Marathe S., Rohondia O. (2020). Hemobilia due to spontaneous rupture of cystic artery pseudoaneurysm: a rare complication of laparoscopic cholecystectomy. *Journal of Case Reports*.

[47] Saldinger P. F., Wang J. Y., Boyd C., Lang E. (2002). Cystic artery stump pseudoaneurysm following laparoscopic cholecystectomy. *Surgery*.

[48] To K., Lai E., Lai E. C., Chung D. T., Chan O. C., Tang C. (2018). Cystic artery pseudoaneurysm with haemobilia after laparoscopic cholecystectomy. *Hong Kong Medical Journal*.

[49] De Molla Neto O. L., Ribeiro M. A. F., Saad W. A. (2006). Pseudoaneurysm of cystic artery after laparoscopic cholecystectomy. *International Hepato-Pancreato-Biliary Association*.

[50] Choudhary A., Barakat M. T., Higgins L. J., Banerjee S. (2016). Choledochoscopic identification of a hepatic/cystic artery pseudoaneurysm in a patient with hematemesis after laparoscopic cholecystectomy. *Digestive Diseases and Sciences*.

[51] Butet Y., Bouras A. F., Truant S., Pruvot F.-R. (2012). Pseudoaneurysm of the cystic artery as a complication of laparoscopic cholecystectomy. *Digestive and Liver Disease*.

[52] Kumar A., Sheikh A., Partyka L., Contractor S. (2014). Cystic artery pseudoaneurysm presenting as a complication of laparoscopic cholecystectomy treated with percutaneous thrombin injection. *Clinical Imaging*.

[53] Petrou A., Brennan N., Soonawalla Z., Silva M. A. (2012). Hemobilia due to cystic artery stump pseudoaneurysm following laparoscopic cholecystectomy: case presentation and literature review. *International Surgery*.

[54] Moses V., Keshava S. N., Wann V. C., Joseph P., Sitaram V. (2008). Cystic artery pseudoaneurysm after laparoscopic cholecystectomy presenting as haemobilia: a case report. *Tropical Gastroenterology*.

[55] Madanur M. A., Battula N., Sethi H., Deshpande R., Heaton N., Rela M. (2007). Pseudoaneurysm following laparoscopic cholecystectomy. *Hepatobiliary and Pancreatic Diseases International*.

[56] Nakase Y., Takagi T., Fukumoto K. (2008). Hemobilia and cystic artery stump pseudoaneurysm associated with liver abscess after a laparoscopic cholecystectomy: report of a case. *Surgery Today*.

[57] Bergey E., Einstein D. M., Herts B. R. (1995). Cystic artery pseudoaneurysm as a complication of laparoscopic cholecystectomy. *Abdominal Imaging*.

[58] Cho N. C., Kim I. Y., Kim D. S., Kim Y. J., Rhoe B. S. (2000). Cystic artery pseudoaneurysm and haemobilia following laparoscopic cholecystectomy. *International Hepato-Pancreato-Biliary Association*.

[59] van Wessem K. J. P., Schelfhout L. J. D. M., Becking W. B., de Smet A. A. E. A., Lange J. F. (1999). Pseudoaneurysm of the cystic artery: a rare complication of laparoscopic cholecystectomy. *International Hepato-Pancreato-Biliary Association*.

[60] Ghosh S. B., Mank G., Lukens F., Colomer A. L. (2010). Cystic artery pseudoaneurysm causing massive GI bleed. *Chest*.

[61] Heyn J., Sommerey S., Schmid R., Hallfeldt K., Schmidbauer S. (2006). Fistula between cystic artery pseudoaneurysm and cystic bile duct cause of acute anemia one year after laparoscopic cholecystectomy. *Journal of Laparoendoscopic and Advanced Surgical Techniques*.

[62] Delgadillo X., Berney T., de Perrot M., Didier D., Morel P. (1999). Successful treatment of a pseudoaneurysm of the cystic artery with microcoil embolization. *Journal of Vascular and Interventional Radiology*.

[63] Morioka D., Ueda M., Baba N. (2004). Hemobilia caused by pseudoaneurysm of the cystic artery. *Journal of Gastroenterology and Hepatology*.

[64] Sibulesky L., Ridlen M., Pricolo V. E. (2006). Hemobilia due to cystic artery pseudoaneurysm. *The American Journal of Surgery*.

[65] Al’aref S. J., Abdel-Rahman H., Hussain N. (2008). Idiopathic cystic artery aneurysm complicated with hemobilia and acute pancreatitis. *Hepatobiliary and Pancreatic Diseases International*.

[66] Thillai M., Sethi P., Narayana Menon R., Puthukudiyil Kader N. (2017). Cystic artery pseudoaneurysm following acute necrotising pancreatitis. *BMJ Case Reports*.

